# Addressing Data Bottlenecks in the Dairy Farm Industry

**DOI:** 10.3390/ani12060721

**Published:** 2022-03-12

**Authors:** Liliana Fadul-Pacheco, Steven R. Wangen, Tadeu Eder da Silva, Victor E. Cabrera

**Affiliations:** 1 Department of Animal and Dairy Sciences, University of Wisconsin-Madison, Madison, WI 53706, USA; tdasilva2@wisc.edu (T.E.d.S.); vcabrera@wisc.edu (V.E.C.); 2 American Family Insurance Data Science Institute, University of Wisconsin-Madison, Madison, WI 53715, USA; srwangen@wisc.edu; 3 Wisconsin Institute for Discovery, University of Wisconsin-Madison, Madison, WI 53715, USA

**Keywords:** data, governance, management, transparency, data sharing

## Abstract

**Simple Summary:**

A better understanding of the current challenges and opportunities regarding data management and data governance in the dairy industry is key to design and define effective data utilization. Thus, a survey was conducted to understand the attitudes of farmers and non-farmers. Respondents strongly agreed that data sharing is a valuable enterprise. They recognized that raw data collected at the farm should be the property of the farmer, and that incentives could motivate farmers to continue, or increase, their data sharing, but most of them were unfamiliar with data collection protocols. Although most farmers are already sharing data, most of them have not signed a data share agreement and feel they do not have data control, once their data are accessed by others. Most respondents exhibited concern about critical data issues, such as ownership, confidentiality, security, lack of integration, and even lack of awareness of the importance of data integration. Farmers indicated that they would be encouraged to adopt a new technology if it is easy to implement and has the potential to improve herd or farm management and profit, whereas they would be discouraged if the technology is expensive, difficult to use, or they do not have clear information about its use.

**Abstract:**

A survey to explore the challenges and opportunities for dairy farm data management and governance was completed by 73 farmers and 96 non-farmers. Although 91% of them find data sharing beneficial, 69% are unfamiliar with data collection protocols and standards, and 66% of farmers feel powerless over their data chain of custody. Although 58% of farmers share data, only 19% of them recall having signed a data share agreement. Fifty-two percent of respondents agree that data collected on farm belongs only to the farmer, with 25% of farmers believing intellectual property products are being developed with their data, and 90% of all said companies should pay farmers when making money from their data. Farmers and non-farmers are somewhat concerned about data ownership, security, and confidentiality, but non-farmers were more concerned about data collection standards and lack of integration. Sixty-two percent of farmers integrate data from different sources. Farmers’ most used technologies are milk composition (67%) and early disease detection (56%); most desired technologies are body condition score (56%) and automatic milking systems (46%); most abandoned technologies are temperature and activity sensors (14%) and automatic sorting gates (13%). A better understanding of these issues is paramount for the industry’s long-term sustainability.

## 1. Introduction

It has been well documented that the development and application of advanced data processing and analytics can have a positive impact on dairy production systems [1,2,3,4]. While this seems straightforward, the implementation of such procedures into production systems is fraught with a number of challenges, including concerns about data collection, data sharing, data ownership, data privacy, and other issues [5]. In an effort to develop a broader understanding of how individuals within the dairy industry (both farmers and other participants) perceive efforts to modernize the collection and use of data, generated on farm, to improve farm practices, a survey was designed as a broad ranging query to, effectively, ‘take the temperature’ of those that would potentially participate in such a system, and attempt to quantify their perspectives on the implementation of a number of data-centric approaches to improving on-farm practices.

Data are a management asset. Valuable information from data can be extracted and used to improve management and optimize decisions [4,5,6,7]. Despite the fact that the digitalization of agriculture benefits the entire dairy industry, concerns about data collection, data sharing, data ownership, data privacy, among others, have risen [5]. These concerns are connected with each other and will have an impact on the willingness of the farmer to share their data and to adopt new technologies [8,9]. One of the important aspects of data sharing is to create an aggregate collection, which can be more effective for analyses and adding value, compared to data at the individual level [10,11]. In addition, the potential value created from data will increase as the sharing participants increase [11] and could possibly lead to new business models that involve data collection by farmers and transformation companies, looking to create value from that data [4,12]. These business models are currently lacking, and the lack of benefits when sharing their data is one of the farmers’ primary concerns [9]. As data are seen as a commodity, the implications of data ownership and how that ownership interacts with newly adopted technologies need to be clarified and discussed to build balanced business models [7]. Another challenge that emerges with data sharing is data privacy. The establishment, adherence to and communication of practices tht preserve data ownership and privacy are complex and difficult to address, because there are currently no legal frames to address this type of data exchange [8,10,12]. Even though there exist voluntary agricultural data codes of practice that may help clarify and understand some of the data contracts, they seem not to be enough [13,14]. Unlike data belonging to individual people, there are no existing laws protecting data belonging to farms (e.g., GDPR [15]). It has been suggested that the focus on data ownership should be to move away from the question of who owns the data, to a more amiable discussion on transparency and credible data management, on an international level, and into developing best practices and goals of data governance and data management, to create and guarantee the benefits of data sharing [8].

Creating awareness within the farmers, in terms of data collection, data sharing and data governance concerns, as well as emphasizing the importance of transparency, are the foundations to guarantee more effective data management practices [9]. Among the objectives of the Dairy Brain Coordinated Innovation Network (CIN) (https://dairybrain.wisc.edu/coordinated-innovation-network/, accessed on 3 January 2022) is to address multiple aspects of data bottlenecks that could disrupt the progress in data-driven dairy management [14]. The Dairy Brain CIN is an industry-wide stakeholder group that was established in September 2019, succeeding an initial project Advisory Committee. Currently, it enlists more than 100 members, composed of worldwide dairy industry professionals, policy makers, and academicians, who provide insights and guidance to the University of Wisconsin-Madison Dairy Brain project [14]. The CIN role is to raise awareness, create guidelines regarding data in the dairy farm industry, and facilitate the process of exploring some of the critical issues around technological adoption and current bottlenecks, related to data management in the dairy industry. To support this process, a survey study was designed with the input of the CIN members, the Dairy Brain team, and the University of Wisconsin survey center. The objective of this paper was to use that survey to explore the mindset of farmers and non-farmers, regarding data sharing, data governance and adoption of data-driven innovations.

## 2. Materials and Methods

### 2.1. Survey

The survey consisted of 3 sections: (1) protocols that oversee the collection, sharing, ownership, and security of data; (2) interest in the use, access, transformation, and integration of farm data; (3) the adoption of decision support tools and other data-driven technologies. These sections covered the main key topics related to data in the dairy farm industry. The questionnaire consisted of 74 questions, most of them coded as multiple choices. The survey was delivered in an electronic format, which allowed us to tailor some of the questions depending on whether the respondent identified as a farmer or as a non-farmer. There was no requirement to answer all the questions (i.e., respondents could skip one or more questions), and all the responses were confidential. The survey was approved by the Education and Social Behavioral Science Institutional Review Board (ED/SBD IRB) of the University of Wisconsin-Madison.

The online questionnaire was distributed to a convenience sample of dairy farmers and other dairy industry stakeholders (consultants, vendors, extensionists, academicians, and researchers). The invitation to complete the survey was delivered via the internet and distributed through available sub-networks from the CIN [14]. A request was submitted to each member of the Dairy Brain CIN to complete the survey and/or resend the invitation link to their own sub-networks of dairy farmers and industry stakeholders. In addition, the questionnaire was also distributed on social media channels such as LinkedIn, Facebook, and Instagram. Data were collected during the first half of 2021. The questionnaire was generated using Qualtrics software (Qualtrics, Provo, UT, USA) [16].

### 2.2. Statistical Analysis

Analyses primarily contrasted responses between farmers and non-farmers to evaluate if there were different mindsets among these two groups. Analyses were also contrasted by age categories of the respondents. Age categories were adapted from the Census of Agriculture 2017 [17] into 4 subgroups (<=33, 40–50, 51–62 and >=63 years). Unless there was a significant difference according to the Chi-Squared test or the Fisher’s exact test [18] (*p* < 0.05) between farmer and non-farmer groups or between age groups of the respondents, results are reported for the whole population of respondents without group distinctions. Questions only applicable to either the farmer or non-farmer group were reported as such. Responses are reported as count, frequency, or percentages. Likert-type responses [19] based on four options (‘yes, always’, ‘in most situations’, ‘in some situations’ and ‘no, never’) were assigned a rank score from 1 (‘yes, always’) to 4 (‘no, never’) and five-point scale (‘not at all’, ‘a little’, ‘somewhat’, ‘very’, and ‘extremely’) were scored from 1 (‘not at all’) to 5 (‘extremely’) to calculate a mean response value that indicates an overall sentiment score (i.e., attitude score) for each of the different factors. Data manipulation and analysis were performed using Qualtrics software (Qualtrics, Provo, UT, USA) [16].

## 3. Results

### 3.1. Demographics of Respondents

There were 169 completed questionnaires. Of these, 73 were from farmers and 96 from non-farmers. Of the farmers, 47 were identified only as farmers and the rest (26) were identified as farmers along with another role (consultant, vendor, extensionist, researcher, other, or a combination of these). The non-farmer group self-identified as consultants (40), vendors (25), extensionists, academics, or researchers (16), or other (15).

**Figure A1:**
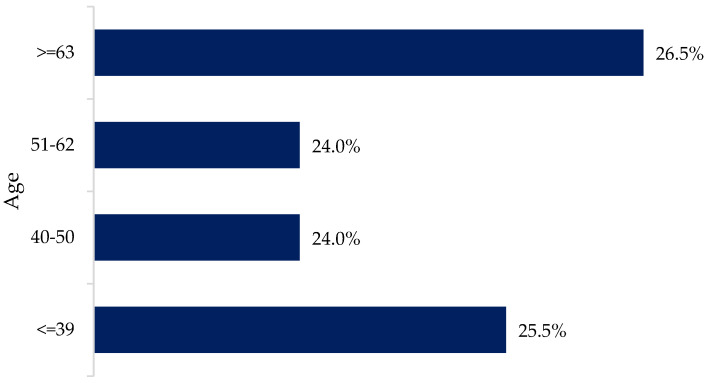
Categories of the respondent’s age.

**Figure A2:**
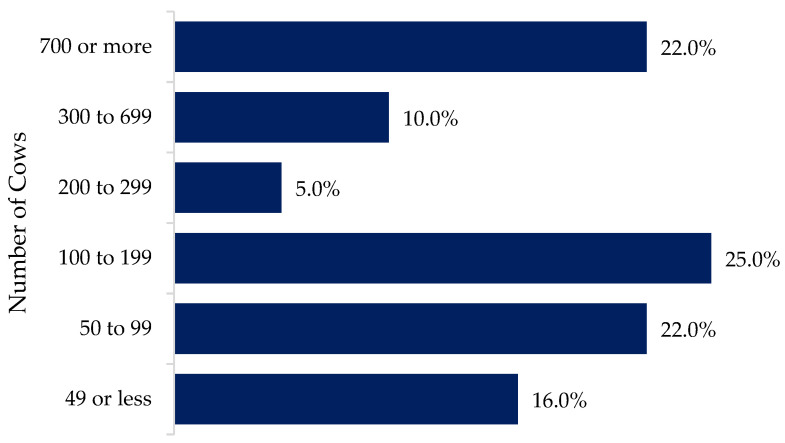
Herd size of the farmers respondents.

The average age of the respondents was 50.8 years old (ranging from 22 to 89 years old) for both groups and there were no statistically significant differences between farmers and non-farmers with relation to age (Figure A1; see Appendix A). The average herd size of the farmers farms was 100–199 cows; grouping of herd size was also evaluated for explanatory power in responses (Figure A2; see Appendix A). Most of the respondents were in the USA (86.4%).

### 3.2. Survey Responses by Sections

#### 3.2.1. Data Collection Protocols, Data Sharing, Data Ownership, and Data Security

- Data collection protocols

When discussing data exchange and data collection, the knowledge of the existence of data collection protocols or animal data exchange standards on dairy farms is key. The survey asked about the familiarity with five existing protocols, which oversee data collection protocols and animal data exchange standards:

- International Committee on Animal Recording (ICAR) [20];
- European Union’s General Data Protection Regulation (GDPR) [15];
- American Association of Bovine Practitioners (AABP) [21];
- National Mastitis Council (NMC) [22];
- Agricultural Data Act of 2018 [23].

Most of these entities were unknown for the great majority of the respondents. The ICAR is known by 12.7% of the farmers and by 38.9% of the non-farmers (*p* < 0.001). Similar results were found for GDPR, where 9.9% of the farmers and 22.91% of the non-farmers responded that they know this entity (*p* = 0.03). This unfamiliarity was significantly lower for the >=63 age group (*p* = 0.04) than the other age groups. A small percentage of farmers and non-farmers (9.9% and 13.7%, respectively) are familiar with the Agricultural Data Act of 2018. The AABP was known by 42.3% of the farmers and 46.3% of the non-farmers. From all these five data collection protocols and animal data exchange standards, the NMC was the most familiar to farmers (60.6%) and non-farmers (51.6%).

- Data sharing

Chain of custody is the concept that farmers always know who has access to their data, at what time, and for what reasons. It is also a critical component of data governance. We asked farmers if they have 100% control over the chain of custody of their data. Most of them (65.7%) responded that they do not have such a control (Table 1). The remaining farmers (34.3%) answered that they believe they have 100% control of their data.

All respondents were asked if there is a significant value or benefit in sharing data outside the farm or organization. Overall, most of the respondents (91%, total) agreed that there was a benefit to sharing data. However, this sentiment was more prevalent in non-farmers (95.8%) compared to farmers (84.5%; Table 1); a statistically significant difference between the two groups (*p* = 0.014). In terms of the proportion of respondents believing that there is a potential for the monetization of the data from the farm, there were similar responses reported (64.3% total, 63% of non-farmers, 37% of farmers); however, in this case, the difference between the two groups was not significant (*p* = 0.051).

A series of four questions were posed to investigate perspectives on business models regarding farmers’ data sharing and vendors’ data added value:

- Should companies pay farmers for their raw data?
- Should farmers pay for the aggregation and added value to the data?
- Should companies reduce the price of technology IF farmers are willing to share their data?
- Should farmers receive value added dashboards and tools in exchange for sharing their data?

The respondents were asked to rate the questions under different situations that were assigned a rank score from 1 (‘yes, always’) to 4 (‘no, never’). This allowed us to calculate a mean response value that indicated an overall sentiment score for each of the different factors. Regarding the first question above, (a) ‘Should companies pay farmers for their raw data?’, the average respondent score for farmers and non-farmers was significantly different (2.06 and 2.42, respectively; *p* = 0.006). For question (b), ‘Should farmers pay for the aggregation and added value to the data?’, the average score was also significantly different among groups, and it was 2.97 for farmers and 2.53 for non-farmers (*p* < 0.001). When asked question (c), ‘Should companies reduce the price of technology IF farmers are willing to share their data?’, the average score was 1.82 for farmers and 2.16 for non-farmers (*p* < 0.013). Finally, in response to question (d), ‘Should farmers receive value added dashboards and tools in exchange for sharing their data?’, both groups agree (average score 1.93 and 1.94 for farmers and non-farmers, respectively, *p* = 0.957) that farmers should receive value added tools in exchange for their data.

After asking about data compensation, the next question was about how companies determine the amount of payment. Would it be based on data type (e.g., genetic data could be more valuable than milk production data); data quality (e.g., data with a lot of outliers or missing values is less valuable); or data quantity (e.g., by data point generated or by cow)? There were no differences between farmers and non-farmers in these responses. Most of the respondents agreed that payment should be based on data quality (90.3%; Table 1), followed by data quantity (78.7%) and data type (78.5%).

To better understand the importance of sharing data for decision making and innovation in dairy farm management, we asked participants to evaluate how beneficial it is to share data with companies, research institutions and other peers. The level of benefit was evaluated as a rank score from 1 (‘not at all’) to 5 (‘extremely’). The overall sentiment score for sharing data with companies, research institutions and peers was significantly higher for non-farmers than for farmers. When asked to score the benefit of sharing data with companies, the average score was 3.91 for non-farmers and 3.33 for farmers (*p* < 0.001). For sharing data with research institutions, non-farmers’ average score was 4.20, compared with 3.71 for farmers (*p* < 0.001). Finally, when sharing data with other peers, non-farmers’ average score was 3.70 and farmer score was 3.29 (*p* = 0.03)

**Figure 1:**
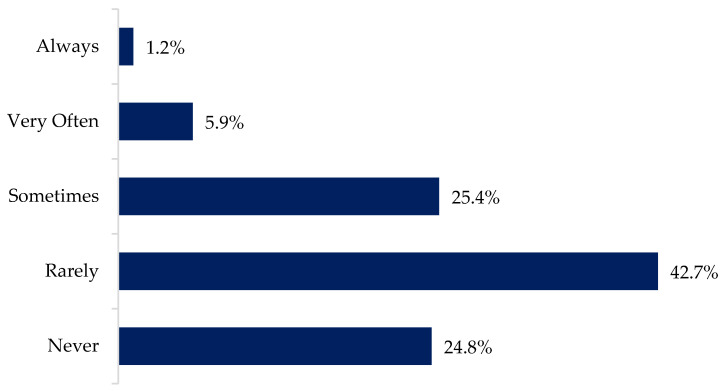
Percentage of respondents that read the terms and conditions of software or technology licenses before clicking “I agree”.

Data sharing agreements are another essential aspect of data sharing and data privacy. The terms and conditions are the cornerstone to understand not only the aspects of the agreement, but also how data privacy is being guaranteed or protected. Results showed that most of the respondents (67.5%) never, or rarely, read the terms and conditions of software or technology use before agreeing to it. Another quarter of the respondents (25.4%) do it only sometimes. Only 7.1% of the respondents indicated to read it either very frequently (5.9%) or always (1.2%) (Figure 1).

From all the respondents, 55% report sharing data outside of their farm or organization. However, from all farmers, 59% indicate that they have not and 22% indicate they do not know if they have actually signed a data sharing agreement during the past 5 years. Only 19% of farmers are certain that they have signed a data sharing agreement within the last 5 years.

**Figure 2:**
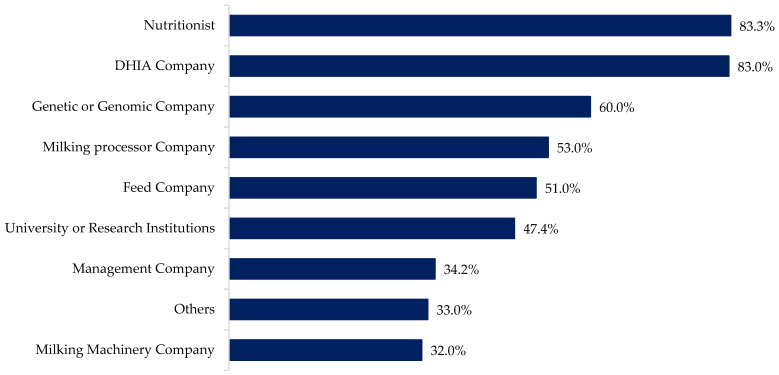
With whom outside the organization farmers are sharing their data.

Data sharing is common within the dairy farm industry; for example, the genetic improvement program (e.g., Dairy Herd Improvement Associations (DHIA) and Council on Dairy Cattle Breeding (CDCB)). Therefore, to follow up on the question about data sharing outside the organization, we asked with whom the data is being shared. From those farmers who share farm data, 83.3% indicate they share it with a nutritionist, 83% with the DHIA, 60% with a genetic or genomic company, and 53% with a milking processing company (Figure 2).

**Figure 3:**
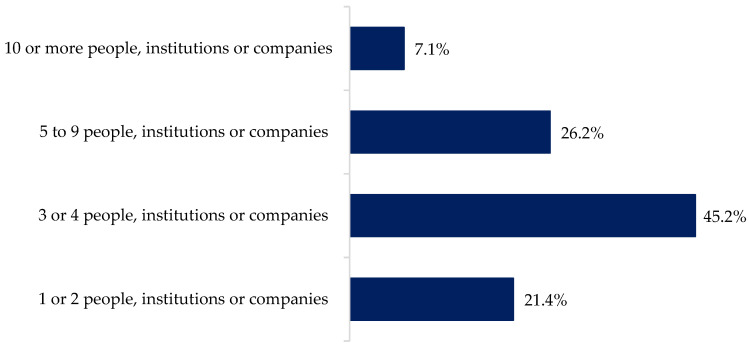
With how many people, institutions, or companies’ farmers are sharing their data.

The type of data these farmers are sharing are milk production data (97%), herd management and feeding data (87%), DHIA data (85%), health data (85%), genetic or genomic data (69%), milk processor data (67%), economic or financial data (61%), and some other data, such as land management (5%). The next question was about the format in which they share their data. A large majority of farmers (93%) share their data through electronic files, whereas almost half of them (49%) still share data on paper, and 9% of them share data verbally. They share data with 1 or 2 (21.4%), or 3 or 4 (45.2%) people, institutions, or companies (Figure 3). Additionally, when asked if they know how to stop the data sharing process, 62% of farmers indicate that they know how, whereas the other 38% of farmers indicate that they do not know how they would stop the data sharing process.

- Intellectual property, data exchange and data management challenges

For the purposes of this study, intellectual property includes inventions, creations, or other works of intellect that may be protectable by patent, copyright, trademark, and trade secrets law. We asked farmers if they believe there are any people, institutions, or companies using data generated on their farm to create models or tools that could be considered intellectual property. A quarter of the farmers (24.7%) believe that there are people, institutions, or companies who are developing models or tools using their data that would warrant intellectual property, whereas 31.5% of them do not know if that is the case, and the remaining 43.8% of farmers do not think that would be the case.

When we asked the question of who owns the raw data collected at a farm, there were no differences in responses between farmers and non-farmers, nor among age categories. Overall, 52% of all respondents state the raw data collected at a farm belongs only to the farmer, whereas 36% of the respondents think it belongs to both the farmer and the company collecting the data, compared to only 8% of the respondents saying that it belongs only to the company collecting the data. Only 4% of the respondents indicated that the ownership would be of someone else, such as the one who pays for collecting the data or the government. Further, 90% of all the total respondents, (97.3% of the farmers and 84.2% of the non-farmers; *p* = 0.008) think that the companies should pay the farmers for their raw data, if the companies are using the data to make money.

To the question of who should own the intellectual property (i.e., rights of inventions, creations, or other works of intellect that could be protected by a patent, copyright, trademarks, or trade secrets), developed by models or other analyses by a university or research institution with farm data, 50.9% think these rights should be shared between researchers and farmers, 29% think the rights should be of the university or research institution, 8.9% the farmer, and 6.5% the individual researcher. About 4.7% of all respondents thought the intellectual property should belong to someone else—being shared between the university and the researcher, those who have made significant contributions to the final product, or no-one (publicly available).

There was a significant difference between farmers and non-farmers, with respect to who should own the intellectual property when the development is performed by a company. Of all the farmers, 59% think it should be shared between the farmer and the company, 22% think it should be only the company, and 16% think it should be only the farmer. Contrastingly, of all the non-farmers, 48% think it should be shared between the farmer and the company, 45% think it should be only the company, and only 6% think it should be only the farmer (*p* = 0.008). Interestingly, 3% of farmers and 1% of non-farmers think that the intellectual property should belong to someone else, such as those who made a significant contribution to the end-product or the company who provides added value to the data.

There was a significant difference between farmers and non-farmers responding to the question of monetary compensation, if farmers were exchanging data for services from a company or researchers. Whereas 58% of farmers believe that companies or researchers should pay additional compensation to the farmer, only 20% of non-farmers think so (*p* < 0.001). Further, 75% of non-farmers think that no additional compensation is needed, whereas only 39% of farmers believe that (*p* < 0.001).

- Perception of data management challenges

**Figure 4:**
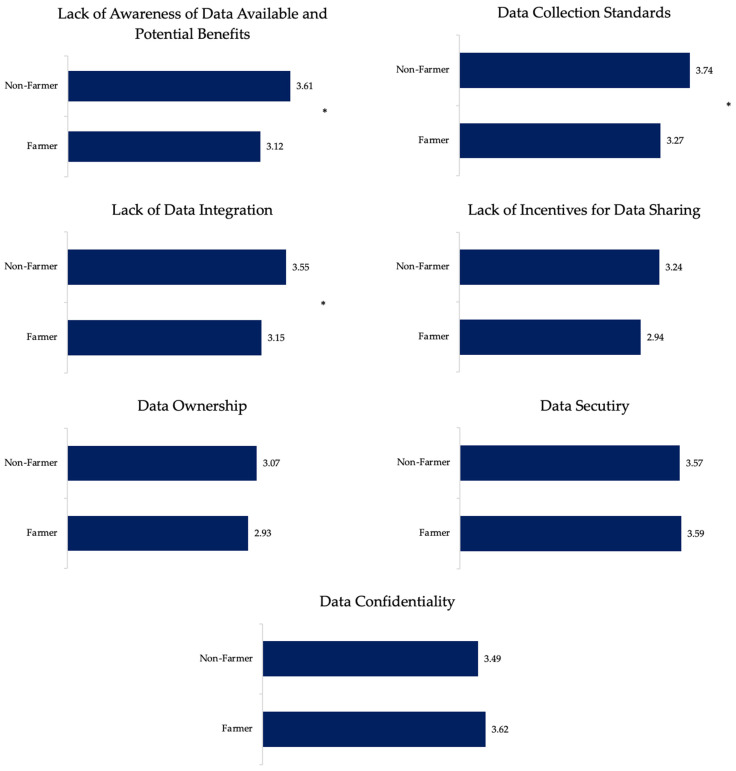
How concerned farmers and non-farmers are about different data management challenges. Level of concern was ranked from 1 to 5, where 1 was ‘not concerned at all’ and 5 was ‘extremely concerned’. The numbers represent the mean sentiment score of farmers and non-farmers of each data management challenge and significant differences (* *p* < 0.05) are indicated.

To better understand the perception of data management challenges, we asked how concerned respondents were about different issues related to data farm management (Figure 4). The level of concern was evaluated as a rank score from 1 (‘not at all’) to 5 (‘extremely’). Regarding data ownership, non-farmers’ and farmers’ average score was similar. However, farmers seem to be more concerned about data confidentiality than non-farmers; farmers were significantly less concerned about data collection standards when compared with non-farmers (Figure 4).

The next question was about data sensitivity, which is linked to data security, or the potential risk entailed if those data were exposed. Both farmers and non-farmers classified the sensitivity of different data types on the farm similarly. The percentage of total respondents who classified the following data types to be “extremely sensitive” were as follows: economic or financial (53%), herd health (13%), milk processor (8%), herd management (8%), other (6%), milking (2%), and feeding data (2%). They listed, among other, “extremely sensitive” data: environmental sensor data, environmental assessment data, individual animal identification data, soil and crop data, animal welfare data, farm or farmer identification data, animal mortality data, farm partnerships, and taxes data.

In order to evaluate concerns around data confidentiality, we asked the respondents to evaluate their confidence that their privacy was sufficiently maintained, when sharing data with companies, universities, and research institutions. Responses indicating the respondents’ level of confidentiality were assigned a rank score from 1 (‘not at all’) to 5 (‘extremely’). There was a significant difference between farmers and non-farmers regarding data confidentiality, when data was shared with research institutions, with an average score of 2.95 for farmers and 3.40 for non-farmers (*p* = 0.006). Similar results were found when sharing data with universities, where the average score for farmers was 3.03 and 3.49 for non-farmers (*p* = 0.006) and with companies, the average score was 2.44 for farmers and 2.87 for non-farmers (*p* = 0.006).

#### 3.2.2. Data Uses, Data Access, Data Transformation, and Data Integration

One of the main objectives of the Dairy Brain project is to integrate data from different sources in real time, to develop data-driven decision support tools [2]. The questionnaire contained a series of questions regarding the process of data integration and its usages. Results showed that, overall, 73.4% of the total respondents used transformed data for decision making. However, decomposing this into farmer and non-farmer groups revealed a significant difference (61.6 vs. 82.3%, respectively; *p* = 0.001; Table 2). On the other hand, 23.3% of farmers indicated they do not use transformed data for decisions, whereas only 9.4% of non-farmers indicated that. The remaining 15.1% of farmers and 8.3% of non-farmers indicated that they do not know if they use this type of data.

Less than one-third (30%) of farmers responded that they are willing to pay for access to transformed data, collected on their farm, while about 27% of farmers indicated they already pay for such a service. From those farmers who pay for access to the transformed data on their farm, 66.7% believe that the price they pay is fair, whereas 93% of those respondents who charge for accessing transformed data think that the price is fair. From all respondents, 15% sell access and 27% have considered selling access to transformed data, for predictive models, analytics, or tools to support decision making to others.

**Figure 5:**
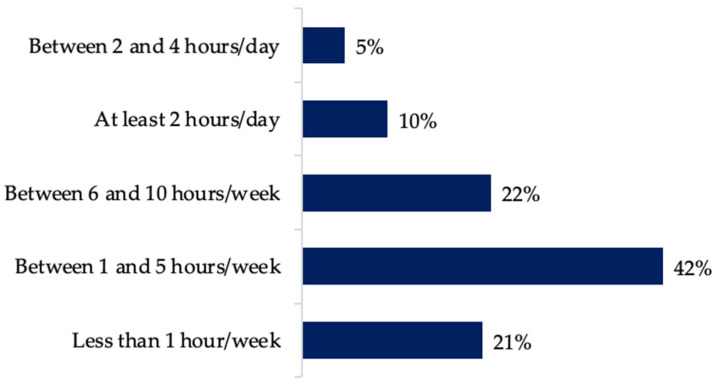
Time farmers spend managing data and/or performing data analysis.

Most farmers (63%) rely on data collected from the farm for daily decision making. However, when asked how much time they spend analyzing data (i.e., retrieving, looking at, or evaluating data), 42% said they spend between 1 and 5 h per week and 21% said they spent less than an hour a week (Figure 5). This means that at least 62.5% of farmers spend an hour a day or less exploring the data to help in the decision-making process.

There were different questions to address data integration and its usage:

- Currently, do you integrate data from different data sources at the farm or at your organization or research institution? And if yes, how is the integration done?
- How useful is data integration for the decision-making process at the farm or for the development of tools at your organization or research institution?
- If you are not currently integrating data, why not?

In terms of integrating data at the farm, 61.6% of the farmers and 70.4% of non-farmers declared that they were carrying out some type of data integration on the farm or at their organization or research institution. In terms of benefits, farmers and non-farmers responded that using integrated data from different data sources is useful (68.4% and 69.7%, respectively). The next question was to evaluate how valuable the use of integrated data is in providing a better understanding of the past or current situation on a farm, to develop management tools to help the decision-making process. The reported value was assigned a rank score from 1 (‘not at all’) to 5 (‘extremely’). There were significant differences between farmers and non-farmers; the average score for using data integration to understand past situations was 3.59 for farmers and 3.91 for non-farmers (*p* = 0.002). Similar results were found for understanding current situations on the farm, where the average score was 3.92 for farmers and 4.29 for non-farmers (*p* = 0.001). In addition, there was also a significant difference between farmers (3.92) and non-farmers (4.43) regarding how valuable data integration can be for the development of decision support tools (*p* < 0.001). Regarding how the integration is done, most of the farmers are doing manual integration (46.8%), whereas most of the non-farmers performing data integration are doing so using software (53.1%). The main reasons why the respondents are not currently integrating data is the lack of required software (81.5%) and lack of time to do it (73.1%).

Additionally, we asked farmers if they use benchmarking, defined in this study as a process of comparing the performance of a farm to a high-performance farm. The point of benchmarking is to identify internal opportunities for improvement. We found that 71.2% of the farmers, at least once per quarter, check and compare their results with those of other farmers through benchmarking.

#### 3.2.3. Technologies and Decision Support Tools: Adoption and Usage

**Figure 6:**
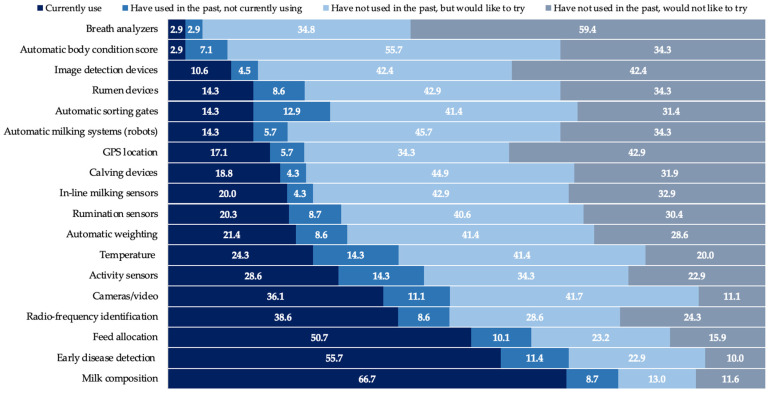
Use of technologies on dairy farms. The numbers inside the bars represent the percentage of responses for each usage category by type of technology.

Survey respondents were asked about their usage of 18 technologies (Figure 6). Answers can be broken down into those that currently use a specific technology and those that do not. Based on this breakdown, the three most commonly used technologies were milk composition, early disease detection and feed allocation. The least utilized technologies listed were breath analyzers, automatic body condition score, and image detection devices.

For those farmers that indicated they had not used a technology, they were also asked to indicate if they would or would not be interested in trying it out on their farm. This allowed us to further decompose these responses, to indicate general interest in the individual technologies. In general, the results showed that interest in trying new technologies (adoption) was generally high, across all options mentioned (on average, 41.2% of respondents not currently trying a technology indicated they would be interested), with the greatest adoption interest found in automatic body condition score (55.7% of respondents, on average, who indicated they had not tried this technology, but would like to; Table 3), automatic milking systems, and calving devices (Figure 6). On the contrary, those technologies with the least interest included breath analyzers, GPS location and image detection devices, for behavior, conformation, or others (Figure 6).

Those that indicated that they have used a given technology were asked to indicate if they are still using (sustained use) or are no longer using (abandoned) this technology. In general, the overall rate of abandonment across all technologies was relatively low (10.4%), suggesting once a technology is in place, farmers are likely to continue utilizing it. The technologies with the greatest rates of abandonment included temperature and activity sensors (14.3% have used, but don’t currently use it) and automatic sorting gates (12.9%).

Farmers were asked to rate the extent to which varying factors either encouraged or discouraged adoption of new technologies on their farm. Responses indicating the respondents’ level of encouragement or discouragement were assigned a rank score from 1 (‘not at all’) to 5 (‘extremely’). Results indicated that respondents were overall most encouraged to adopt a new technology based on ‘ease of implementation’ (average respondent score = 2.76) (Table 3) and the potential of the technology to ‘improve herd or farm management and profit’ (2.75). The ‘option to share the information with different personnel’ scored lowest (1.97) for encouragement. There was less variability in the discouragement assessment, with the highest score (least discouragement) expressed for difficulty of use or operation and lack of information (both with a score of 1.69), and the lowest score (greatest discouragement) coming from initial cost of the technology (1.43).

Farmers were asked to classify how useful they found several reporting mechanisms of technologies and/or decision support tools (i.e., dashboard, a notification, a text message, a daily report, or an email). The responses were assigned a rank score from 1 (‘not at all’) to 5 (‘extremely’), which was used to calculate a mean response score for the group and compare the ‘usefulness’ of the different mechanisms. The mechanism with the highest score was a daily report (2.50), followed by a text message (2.32). ‘An email’ received the lowest (least favorable) score (1.93) of the mechanisms listed.

Regarding effective and more user-friendly communication from these technological systems, 31.5% of farmers say they do not receive any type of notification via email, cell phone or apps from data systems (87% of these farmers do not receive notifications because the systems they use do not have this functionality). In addition to these, 12.3% of respondents said they only receive some kind of notification when something stops working. The remaining 56.2% farmers said they received some type of notification (i.e., less than one per day to three or more times per day). When farmers were asked if they currently use a decision support tool to aid farm management, 47.3% answered yes and 52.7% answered no. Evaluating responses in the context of age groups, the middle age group (40–50 years) reported significantly (*p* = 0.04) less adoption of decision support tools when compared to either younger (<40 years) or the oldest (>62 years) age groups.

Farmers that are currently using decision support tools to aid farm management were asked for which decisions they are currently using the decision support tools; ‘when to cull a cow’ was reported as the most common decision (82.9% of respondents answered yes), followed by ‘when to flag cows with health issues’ (77.1%) and ‘when to change reproduction protocols’ (71.4%). The decision where support tools were least commonly applied was ‘when to change milk protocols’ (44.1%).

## 4. Discussion

### 4.1. Management and Control of Data

The results of this survey reaffirm and clarify some insights about different aspects of data management on dairy farms. Data sharing is an important aspect to improve farm management, quality, and quantity of the end-product [24]. Agreements play a crucial role in facilitating data sharing, by defining the terms around sharing and data privacy. The documented terms and conditions of data management are the cornerstone to guaranteeing and communicating aspects, not only of how the data is shared, but also how privacy is being guaranteed and protected. However, our results shown that 67% of farmers never, or rarely, read the terms and conditions before agreeing to them, suggesting these terms are not fully understood by, or conveyed to, those producing the data (in this case, farmers). Other studies had reported that up to 97% of people agreed to the terms and conditions without reading them, whereby the sentence “I agree/read the terms and conditions” has been cataloged as one of the biggest lies on the internet [25,26]. In an independent survey of 1000 producers, among 17 different industries, results showed that 74% of the respondents did not know much about the terms and conditions [9]. In our survey, when asked to rate if they read before clicking ‘I agree’ to the terms and conditions, on a scale from 1 (never) to 5 (always), the average score was 2.10 for farmers and 2.22 for non-farmers. Similar results were reported by Wiseman et al. [9], where dairy respondents had a score of 2, where the scale was 1 (don’t know at all) to 5 (know very well), suggesting that farmers are even less familiar with the terms and agreements for data sharing than their counterparts in other industries.

Wiseman et al. [9] reported that farmers who had a better understanding of terms and conditions agreements were more willing to share their data. Indeed, results of this survey showed that although farmers and non-farmers think that sharing data is important, 81% of the farmers surveyed reported having not signed, or not knowing if they had signed a data sharing agreement. Additionally, 38% of the farmers responded that, if desired, they do not know how to stop the data sharing agreement. Overall, the perception of respondents is that farmers do not have control over their data (i.e., chain of custody). These results suggest an underlying mistrust that exists when it comes to sharing data that is not addressed by communicating the terms via existing service agreements, and this lack of transparency and communication can likely reduce the perceived added value of the data [11].

Our results suggest that farmers considered that sharing data with research institutions and peers is potentially more beneficial than sharing with companies. Similar results were reported by Zhang et al. [27]. However, our results showed that respondents do not feel confident about companies, universities or research institutions protecting their data. The average scores were significantly lower for farmers compared with non-farmers. Similar results were reported by Wiseman et al. [9], who found that 62% of the respondents revealed no trust at all or too little trust in the service providers maintaining the privacy of their data. There also seems to be a difference among livestock industries, as the poultry industry reported the lowest trust, whereas the dairy reported an intermediate trust level [9].

### 4.2. Value of Farm Data

Most farmers and non-farmers agree that the raw data produced on a farm is owned by the farmer. Most of the respondents (90%) thought that farmers should be compensated when the companies are financially benefiting from the data. Studies suggest that one of the steps to address this value imbalance is to concede benefits to the users (i.e., farmers) in forms, such as reducing costs or engaging them in a participatory economic culture [11,28]. To better understand the value of the data, Foy [29] compared the data with rare-earth minerals, for which their market value depends on extraction, refinement, and transformation. Likewise, the value of an individual point of data is low, but having a system in place to make use of millions and trillions of data points has an important value, both on and off the farm [29].

The results of our survey demonstrate that 27% of farmers are currently paying a ’fair’ price for the transformation of data that originates on their farm, and an additional 30% demonstrated a willingness to pay for such a service. It has been reported that data integration is lacking but it is critical to improve decision making, management, and welfare [30,31]. Most of the farmers (85.5%) considered integration can be very valuable for the development of decision support tools. Together, these results suggest that there exists strong potential involvement from the dairy community in a data-driven economy that could be mutually beneficial, for both the farmers and those providing value-added service to enhance their data. Wysel et al. [11] proposed that data might become a commodity that could be exchanged as traditional assets, which could then be used for value generation through proprietary development.

Despite those farmers being fully aware about it, data integration is not an automatic task in farms, which can be corroborated by the fact that 46.8% of farmers are doing the integration manually. In addition, non-farmers are very concerned about the lack of awareness and data integration on dairy farms and their potential benefits. Among the reasons that could explain why at least 63% of farmers spend less than an hour a day exploring the data to help the decision-making process, the high workload and lack of time, in addition to the lack of tools and software that would provide farmers with relevant information, in a user-friendly way, could be factors.

### 4.3. Technological Adoption

When the questions were about current usage and adoption of technologies, results indicated a strong correlation between respondents who currently use and those who demonstrated interest in the following technologies: cameras, milk composition measurement devices, and radio frequency identification technologies. In contrast, one of the technologies with the second highest reported current use, early disease detection, was highly correlated with a lack of interest from non-users. A similar pattern was observed for feed allocation and calving devices technologies. Positive and negative correlations among the current usage and the interest of adopting or not was also reported by Gillespie et al. [32]. Another factor that has been reported to potentially have a variable effect in technological adoption is age [33,34,35,36,37]; where some studies report that increasing age correlates with a decrease in adoption [34,35,36], others found age had no effect [37,38]. Tamirat et al. [35] found that farmers under 50 years have a higher tendency to adopt when compared with older farmers. When evaluating the adoption of decision support tools in this survey, the middle age group reported significantly less adoption when compared to either younger or the oldest age groups. However, the difference in how ages were categorized in the different studies makes direct comparisons difficult. To address this issue, and in order to have a more representative indicator, Burton [39] suggested using an age index of the family members as a better indication of the current situation of the farm.

Some of the results of our survey reaffirm certain practices or concepts about farm data management and technology adoption. Common among farm practices are benchmarking practices across farms and the need of keeping historical data. Regarding technologies, the ease of its implementation is a must for the adoption. A literature review of technology adoption [38] also reported that the primary drivers of adoption are the observed usefulness and the ease of use. More recently, Baldin et al. [40] discussed the sustainable adoption of integrated decision support tools and highlighted the importance of easy implementation, but also underlined the willingness of the dairy industry to work together to coordinate a data sharing process that benefits the entire system. In addition, lack of transparency and confidence in data security are also hurdles for adoption technologies [8].

## 5. Conclusions

The potential for a successful data economy to operate within the dairy industry exists and is demonstrated by the overall sentiment that there is a positive perception of the ability to add value to data from the farm, as well as a general willingness for technological adoption. This potential is hindered by distrust, in terms of services and privacy protections, along with operational hurdles to the adoption of new technologies. Resolving issues around adoption and trust can facilitate the potential (both practical and economical) for data-driven decision making (and the accompanying data economy), leading to increased operational efficiency, increased profits, and reduced impacts (e.g., animal welfare, production). Further research is needed to explore, in more detail, some of the challenges found in this survey, such as the lack of confidence in institutions. Having a better understanding of the current status and all potential concerns discussed in this paper may help tackle specific issues to overcome these challenges.

## Figures and Tables

**Table 1:** Response summary of the data collection protocols, data sharing, data ownership, and data security.

Question	Farmer Agreeing with Statement, %	Non-Farmer Agreeing with Statement, %	*p*-Value
Knowledge about **data collection protocols ^a^**	27	35	NA ^b^
**Data sharing** out of the farm is worth it	84.5	95.8	0.14
Farmer signed a **data share agreement** in the last five years	19.2	NA ^c^	NA ^b^
Farmer knows who has **access to their farm data**	34.3	NA ^c^	NA ^b^
**Never or rarely read** the terms and conditions before clicking “I agree”	68.5	66.7	0.38
Farmer sole **owner of raw data** collected at the farm	48	55	0.32
Companies or researchers should **pay the farmer for sharing raw data**	68.3	31.7	0.03
**Intellectual property** of a product developed by a university, research institution or company **should be shared** with the farmer, researcher, university, research institution, or company	57.5	45.8	0.06
**Data quality** should determine the amount of payment when **compensating for data**	90.3		NA ^b^

^a^ Simple average of knowledge of the existence of five data collection protocols and animal data exchange standards. ^b^ Not applicable, not a direct comparison. ^c^ Not applicable as question was only for farmers.

**Table 2:** Response summary of the data uses, data access, data transformation, and data integration.

Question	Farmer Agreeing with Statement, %	Non-Farmer Agreeing with Statement, %	*p*-Value
Use of **transformed data** for **decision making**	62	82	0.009
**Currently paying** to **access transformed data** of their farms	27	NA ^a^	NA ^a^
**Price paid** to access their **transformed data** is **fair**	66.7	NA ^a^	NA ^a^
**Charged price** to access the **transformed data** is **fair**	NA ^a^	82.4	NA ^a^
Time exploring **data** for **decision making is** **less than** **1 h/day**	63	NA ^a^	NA ^a^
Using **integrated data** is **useful**	68	70	0.320
**Data integration** is **done manually**	47	34	0.295
Keep **historical** data is a common practice	87	NA ^a^	NA ^a^

^a^ Not applicable as question was not asked to this group.

**Table 3:** Response summary of the technologies and decision support tools: adoption and usage.

Question	Farmer Agreeing with Statement, %
**Automatic body condition score** technologies had the most **interest** to be **adopted**	56
**Abandonment** across **technologies** was relatively low	10.4
**Ease of implementation** is a must for the **adoption** of a technology	69
**Daily reports** are the **preferred reporting** mechanism	44
**‘When to cull a cow’** is the most common **decision** taken from a **decision support tool**	83

## Data Availability

The data presented in this study are available on request from the corresponding author. The data are not publicly available due to privacy restrictions.
